# Determinants of access and utilization of cervical cancer treatment and palliative care services in Harare, Zimbabwe

**DOI:** 10.1186/s12889-019-7355-3

**Published:** 2019-07-29

**Authors:** O. Tapera, G. Dreyer, W. Kadzatsa, A. M. Nyakabau, B. Stray-Pedersen, S. J. H. Hendricks

**Affiliations:** 1 University of Pretoria, School of Health Systems and Public Health, Pretoria, South Africa; 2 Department of Obstetrics and Gynaecology, Gynaecologic Oncology, University of Pretoria, Pretoria, South Africa; 3 Radiotherapy Centre, Parirenyatwa Group of Hospitals, Harare, Zimbabwe; 4 Institute of Clinical Medicine, University in Oslo and Womens’ Clinic, Oslo University Hospital, Oslo, Norway; 5 Sefako Makgatho Health Sciences University, Pretoria, South Africa; 6 University of Fort Hare, East London, South Africa

**Keywords:** Cervical cancer, Access, Determinants, Treatment, Palliative care, Inequity, Health system, Sequential mixed methods, Zimbabwe

## Abstract

**Background:**

Cervical cancer treatment and care services have remained largely centralized in Zimbabwe thereby entrenching inequities to access amongst patients. The objective of this study was to investigate the determinants of access to treatment and care among women with cervical cancer in Harare, Zimbabwe.

**Methods:**

A sequential explanatory mixed methods design was used. In phase 1, three surveys (namely community, patient and health worker) were conducted with sample sizes of 143, 134 and 78 participants respectively. Validated structured questionnaires programmed in Android tablet with *SurveytoGo* software were used for data collection during the surveys. Univariate, bivariate and multivariate logistic regression analyzes were conducted using *STATA®* version 14 to generate descriptive statistics and identify determinants of access to cervical cancer treatment and care. In phase 2, 16 in-depth interviews, 20 key informant interviews and 6 focus groups were conducted to explain quantitative data. Participants were purposively selected and saturation principle was used to guide sample sizes. Manually generated thematic codes were processed in *Dedoose* software to produce final outputs for qualitative study.

**Results:**

Knowledge of causes (*p* = 0.046), perceptions of adequacy of specialists (*p* < 0.001), locus of control (*p* = 0.009), service satisfaction (*p* = 0.022) and walking as a means of reaching nearest health facilities (*p* < 0.001) were associated with treatment or perceptions of access by healthy women. Perceptions of access to treatment amongst health workers were associated with their basic training institution (*p* = 0.046), health service quality perceptions (*p* = 0.035) and electricity supply status in their respective health facilities (*p* = 0.036).Qualitative findings revealed health system, societal and individual factors as barriers to accessing treatment and palliative care.

**Conclusions:**

There are numerous prevailing multi-dimensional barriers to accessing cervical cancer treatment and palliative care in a low –income setting. The findings of this study revealed that heath system and societal factors were more important than individual level factors. Multi-sectoral approaches are recommended to address all the multifaceted barriers in order to improve cervical cancer treatment and palliative care access for better outcomes in resource-limited contexts.

## Background

Cervical cancer treatment and care services are still largely centralized in Zimbabwe. These services include radical surgery, radiation, chemotherapy and palliative care [1, 2]. In the health facilities where treatment is available, costs are exorbitant and the referral systems are challenged to afford women with advanced cervical cancer high level of care. Paucity of treatment and care services for cervical cancer has promoted medical tourism to countries such as South Africa, India, China and Europe by the affluent members of society [2]. This has a negative impact on perceptions, acceptance and utilization of health services in Zimbabwe and results in inequities [3]. Most patients in Zimbabwe rely on out-of-pocket financing as only 10% of the population is on medical aid [3]. Furthermore, years of macroeconomic challenges have also compromised the health service delivery system across the country [1, 2]. Consequently, a paradigm shift is imperative in the Zimbabwean health system if access to cervical cancer treatment and palliative care are to be improved to reduce the associated relatively high mortality and morbidity rates. Health policies to address the challenges faced by patients are crucial in an endeavor to afford them their basic right to health as enshrined in the International Declaration of Human rights [4]. However, determinants of access to treatment and care among women with cervical cancer in their various dimensions: individual, societal and health system levels are less understood in Zimbabwe.

In 2000 Chirenje et al. [5] reported that 77% of women diagnosed with cervical cancer between 1995 and1997 were engaged into treatment using radiotherapy. Pomerai et al. [6] also reported that about 7000 women were diagnosed of pre-cervical cancer and cervical cancer every year and of these only 1300 (19%) received treatment. No recent data exists on the proportions of women with confirmed cervical cancer who have engaged into treatment and care. While the Zimbabwe National Cancer Registry documents all reported cancer cases in the country, some of the cases are not reported due to low diagnostic, surveillance and treatment uptake follow-up capacities [1–3]. Fallala & Mash [7] in their Bulawayo, Zimbabwe study noted that of the 10.8% women who tested positive to visual inspection with acetic acid cervicography (VIAC) 61.1% received immediate treatment while 38.9% were either delayed or referred to a gynaecologist. There is no evidence of a follow-up system to track the 38.9% of women who tested positive to VIAC to ensure or sustain their engagement into treatment and care. Lack of follow-up system of cases predisposes women to high risk of late stage disease presentation which is associated with poor outcomes in low-income settings. Consequently, for advanced cervical cancer palliative care is the only treatment modality applicable. There is impeccable evidence that the provision of treatment and palliative care services among women with cervical cancer is still very limited in Zimbabwe [1, 2].

Zimbabwe formulated two key strategies namely: National Cancer Prevention and Control (2013–2017) and National Cervical Cancer Prevention and Control (2016–2020) to establish a policy framework to drive the cancer agenda [8, 9]. However; due to limited resources, competing priorities and other systemic gaps not much progress has been achieved to improve cervical cancer treatment and care in the country [1, 2, 6]. Despite the prevailing limitations, the Ministry of Health and Child Care and its partners have relatively improved cervical cancer screening, awareness and treatment of pre-cancerous lesions over the past few years. In addition, cervical cancer screening services have also been integrated with HIV/AIDS services in major health facilities across the country [2]. While knowledge about the disease, its risk factors and screening programmes has improved over the past few years, a huge information gap still exist about treatment and palliative care interventions in the country. In a study in Zimbabwe by Pomerai and colleagues [6] several factors were shown to influence knowledge about cervical cancer. These included lack of prioritization of the disease by health workers and Ministry of Health and Child Care, lack of a clear case definition of the disease as well as limited funding from global donors. Our study was envisaged to address some of the knowledge gaps with regards to the key determinants of access and usage of cervical cancer treatment and care in Zimbabwe.

## Methods

### Study design

This study used a sequential explanatory mixed methods design of both quantitative and qualitative research methods to generate evidence [10–13]. The priority of the design was on quantitative nature of the study and the implementation was in a sequential manner . This Study was primarily based on a quantitative design in phase 1, while qualitative methods were used in phase 2 to interpret and explain some of the findings from surveys. In phase 1, analytical cross sectional survey approach was used to collect quantitative data and this study design was used in investigating the relationship between different variables and access to treatment was the dependent variable [11–13]. In this phase three surveys were conducted, that is, community based survey, health facility (patient) based survey and health worker survey. In phase 2**,** qualitative design was mainly used to explain significant, non-significant, outliers and surprising results from the surveys [10, 13]. The findings of phase 1 and 2 were integrated at interpretation stage and a combined analysis was utilized to generate evidence for this study.

### Target population, sample sizes and data collection

The target population for Phase 1(quantitative surveys) consisted of three groups: 1) healthy women 2) cervical cancer patients or survivors (previous history of cervical cancer) aged 25 years or older and 3) health workers directly involved in cervical cancer screening, diagnosis and treatment. The age range was informed by studies and anecdotal evidence from health facilities that have shown the incidence of cancer being higher among women who are older than 30 years [5, 6]. In Zimbabwe cervical cancer screening is recommended for sexually active females between 18 and 65 years old [7] and for that reason it was plausible to recruit participants aged at least 25 years old for this study. Furthermore, throughout the data collection process all women with cervical cancer identified in the selected study sites were at least 25 years. Group 1 consisted of 143 healthy women who were resident in Harare (urban and rural) for at least 1year and were randomly selected. Multistage stratified random sampling was used in which four areas in Harare were selected for community based survey to represent each of the following: high, medium, low density surbubs and rural areas. The three suburbs were selected from a total of 138 suburbs in Harare urban. One of the study areas was a rural community and it was selected randomly out of seven communities in Harare. The Kish grid approach was used to select one respondent in households with more than one eligible respondent as it is the most commonly used technique in surveys in Zimbabwe [14]. Group 2: One hundred and thirty-four (134) cervical cancer patients or survivors with histologically confirmed disease were also randomly selected at Harare and Parirenyatwa hospitals as well as from the Island Hospice and Cancer Centre. Women with cervical cancer were selected from health facilities as they made treatment or review visits (outpatients) or from databases or records (in-patients) regardless of where they were resident. This is because the chosen health facilities were tertiary facilities that treat and care for patients from across the country and for this study it was important to reduce bias by selecting patients regardless of where they stayed. Group 3: For the health worker survey, 78 participants were selected from the health facilities from which women with cervical cancer were drawn. Community survey sample size was based on Dopson sample size calculations [6, 11, 15]. Patient and health worker surveys used census approach of patients and health workers who visited or were stationed at the study sites during the period January–April 2018. Previous year (2017) records of cervical cancer patient flow had shown that about 80 patients had visited the study sites collectively during the same period; January–April. However, a total of 134 women with histologically confirmed cervical cancer were finally enrolled in the study and this improved the precision of estimates. Staff records in the study sites had revealed that about 60 health workers of different specialties relevant to cervical cancer treatment and care were permanently stationed in the study sites. During the study period, 78 health workers were enrolled though some were part-time, volunteer staff and consultants who had not been included in the usual records. Those participants who consented in writing and in the case of patients were also clinically stable to be interviewed were enrolled in the surveys. Staff registers were used as the sampling frame from which health worker participants were selected to avoid bias by selecting from those on duty at the time of the survey. Validated structured questionnaires were used for data collection for surveys [16]. Questionnaires were programmed in *SurveytoGo* software in an Android tablet to allow for electronic data collection.

The Qualitative part of the study (phase 2) was conducted in the same areas where survey data were collected. However, the participants in the two phases of the study were different. A total of 84 participants were purposively selected based on desired characteristics for the study. Six focus groups with an average of 8 participants each were conducted in the communities and health facility (Parirenyatwa Hospital). For in-depth and key informant interviews 16 and 20 participants were enrolled for participation based on theoretical saturation principle [17]. All in-depth interviews and FGDs were conducted in communities and health facilities. In communities, church premises were used for conducting focus groups after permission from church elders was obtained. Key informant interviews were conducted in health facilities and in offices of participants. Interview and discussion guides were used for qualitative data collection and responses were audio-recorded after obtaining written consent.

### Data analysis

#### Quantitative surveys

Data analysis were conducted using *STATA®* version 14 software by the researcher to yield descriptive statistics and to compare and establish the nature of relationships between variables. Univariate analysis was used to generate descriptive statistics and bivariate analysis was conducted to identify significant factors for multivariate logistic regression models. Multivariate binomial logistic regression was conducted to identify individual, societal and health system determinants of access and utilization of cervical cancer treatment and palliative care services in Harare [18].

#### Qualitative study

Transcription and translation of audio-recordings were undertaken by the researcher and his assistants. Transcripts were identified by the unique identifier previously assigned to each participant (and stated by each participant at the beginning of the FGD/interview) rather than by any personal information. Unique identifiers were used to link the interview guide and the interview only after the conclusion of transcription. All in-depth interviews, key informant interviews and FGDs were coded manually line by line by the researcher using *Dedoose software* after creation of codes based on the research questions,literature and survey data. Manually generated thematic codes were processed in the same software to produce final outputs for phase 2. Findings of the phase 1 and the phase 2 were integrated at interpretation stage in which case qualitative findings assisted in explaining and interpreting significant, non-significant, outliers and surprising results from the quantitative study.

## Results

### A. Quantitative results

Table 1 shows that the proportions of untreated patients from high density suburbs (50% vs 44%) (*p* = 0.025), affiliated to protestant churches (18% vs 23%)(*p* = 0.028) and other religions (7% vs 2%)(*p* = 0.006), those whose household heads had no formal education (37% vs 1%) (*p* = 0.017) and were professionals (17% vs 22%) were significantly different from those treated (*p* = 0.038). Proportion of cervical cancer patients who were treated was 69% while proportion of healthy women who had the perception of access to treatment if diagnosed of the disease was 80%.

**Table 1:** Characteristics of 143 healthy women and 134 women with cervical cancer

Participant type	Healthy women *N* = 143	Cervical cancer patients *N* = 134		
Variables	[*N* = 143] (%)	[*N* = 134] (%)	Treated [*n* = 92] (%)	*p*-value*
Province of residence				
Manicaland	–	17(12)	14 (15)	0.193
Masvingo	–	9(7)	8 (9)	0.176
Midlands	–	7(5)	5 (6)	0.871
Matebeleland North	–	1(1)	1 (1)	0.498
Mashonaland Central	–	5(4)	4 (4)	0.671
Mashonaland East	–	24(18)	16 (17)	0.871
Mashonaland West	–	5(4)	3 (3)	0.671
Harare	143 (100)	66(49)	41 (45)	0.217
Residence				
Urban	93 (65)	74 (55)	47 (51)	0.154
Urban_Low density	31(21.7)	3(2)	3 (3)	0.237
Urban_High density	31 (21.7)	67(50)	40 (44)	**0.025**
Urban_Medium density	31 (21.7)	4(3)	4 (4)	0.170
Rural	50 (35)	60(45)	45 (49)	0.154
Age (years)	Mean (35)	Mean (52)	Mean (53)	
25–34	78 (55)	6(4)	4 (4)	0.914
35–44	40 (28)	31(23)	19 (21)	0.313
45–54	22 (15)	41(31)	26 (28)	0.642
55 or more	3 (2)	56(42)	43 (47)	0.180
Ethnicity				
Shona	133 (93)	130(97)	88 (96)	0.170
Ndebele	6 (4)	2(1)	2 (2)	0.336
Other	4 (3)	2(2)	2 (2)	–
Marital status				
Married/co-habiting	98 (69)	52(39)	30 (33)	0.029
Never married	17 (12)	1(1)	1 (1)	0.498
Widowed	13 (9)	59(44)	44 (48)	0.190
Divorced or separated	15 (10)	22(16)	17 (18)	0.341
Religion				
Roman Catholic	24 (17)	34(25)	24 (26)	0.779
Protestant	22 (16)	24(18)	21 (23)	**0.028**
Pentecostal	56 (39)	34(25)	21 (23)	0.316
Apostolic sect	27 (19)	34(25)	24 (26)	0.779
Other	14(9)	8(7)	2 (2)	**0.006**
Education				
Primary	19 (13)	43(32)	30 (33)	0.849
Secondary	100 (70)	75(56)	50 (54)	0.576
Higher	24 (17)	6(5)	5 (5)	0.428
None	0	10(7)	7 (8)	0.924
Household head education				
Primary	5 (3)	16(12)	12 (13)	0.560
Secondary	74 (52)	50(37)	30 (33)	0.096
Higher	54 (38)	14(10)	12 (13)	0.146
Not Applicable	10 (7)	5(4)	37 (40)	0.194
None	–	49(37)	1 (1)	**0.017**
Occupation				
Unemployed	59 (41)	90(67)	60 (65)	0.478
Student	7 (5)	3(2)	2 (2)	0.336
Professional	14 (10)	3(2)	3 (3)	0.620
Police/Military/Security	5 (4)	12(9)	9 (10)	0.137
Trucker/transport business	1 (1)	1(1)	2 (2)	–
General worker	6(4)	1(1)	4 (5)	0.318
Self employed	26 (18)	5(4)	10 (11)	0.572
Vendor	25 (17)	16(12)	2 (2)	0.940
Occupation of household head				
Unemployed	9 (6)	25(19)	15 (16)	0.301
Farm worker	1 (1)	2(1)	2 (2)	0.336
Professional	52 (36)	23(17)	20 (22)	**0.038**
Police/Military/Security	11 (8)	5(4)	5 (5)	0.124
Trucker/transport business	15(10)	1(1)	0	0.137
General worker	5 (3)	0	0	0.246
Self employed	31 (22)	30(22)	18 (20)	0.498
Vendor	7 (5)	1(1)	1 (1)	0.621
Other	1 (1)	47(35)	0	–
Not applicable	11 (8)	0	31 (34)	–
Personal income (monthly) (US$)				
No income	52 (36)	77(57)	52 (57)	0.744
< 200	51 (35)	32(24)	23 (25)	0.653
200–400	24 (17)	19(14)	13 (14)	0.981
430 or more	16 (12)	6(4)	4 (4)	0.914
Household income (monthly) (US$)				
No income	52 (36)	71(53)	50 (55)	0.640
< 600	55 (39)	53(40)	35 (38)	0.597
600–1000	16 (11)	6(4)	4 (4)	0.914
1200 or more	20 (14)	4(3)	3 (3)	0.718
Medical insurance/aid				
Yes	50 (35)	27(20)	22 (24)	0.108
No	93 (65)	107(80)	70 (76)	–
Wealth quintiles				
Poorest	50 (35)	7(5)	6 (7)	0.318
Poorer	22 (15)	32(24)	23 (25)	0.653
Middle	19 (13)	36(27)	23 (25)	0.471
Richer	30 (21)	26(19)	15 (16)	0.179
Richest	22 (16)	33(25)	25 (27)	0.311
Access/perception of access to treatment				
Yes	114 (80)	92 (69)	–	–
No	29 (20)	42 (31)	–	

Bold shows *p* value < =0.05 indicating statistical significance. *The *p*-value is for comparison of treated and untreated women with cervical cancer

Table 2 shows that knowledge of causes of cervical cancer was negatively associated with receipt of treatment by patients (*p* = 0.046) and positively correlated with perceptions of access to treatment by healthy women (*p* = 0.016). However, knowledge of prevention of cervical cancer did not influence uptake of treatment or perceptions of access thereof. Locus of control with regards to cervical cancer was positively associated with uptake of treatment among patients (*p* = 0.009) but was not associated with perceptions of access to treatment among healthy women. Receipt of treatment and perceptions of access to it were not associated with any societal factors. Perceptions of adequacy of specialists were positively associated with uptake of cervical cancer treatment (*p* < 0.001) and perceptions of access to it among healthy women (*p* = 0.013). Service satisfaction was correlated with receipt of treatment services among cervical cancer patients (*p* = 0.022). Walking as a means of reaching nearest health facilities was negatively associated with perceptions of access to treatment for cervical cancer among healthy women (*p* < 0.001).

**Table 2:** Determinants of access and utilization of cervical cancer treatment and care from healthy women and patient surveys

Participant type	^a^Healthy women with perceptions of access to treatment if diagnosed of cervical cancer (*n* = 70)		Cervical cancer patients treated (*n* = 92) [*N* = 134]	
Variables	OR, (95% CI)	*p* value	OR, (95% CI)	*p* value
Individual factors^b^				
Knowledge of prevention	0.34 (0.06 to 1.88)	0.218	2.50 (0.53 to 11.97)	0.248
Knowledge of causes	**6.18 (1.03 to 37.14)**	**0.046**	**0.13 (0.02 to 0.68)**	**0.016**
Perception of availability of treatment services	1.08 (0.36 to 3.23)	0.889	1.89 (0.65 to 5.54)	0.244
Affordability of treatment services	0.50 (0.17 to 1.43)	0.197	1.57 (0.45 to 5.59)	0.480
Locus of control regarding cervical cancer	0.97 (0.39 to 2.42)	0.949	**2.90 (1.30 to 6.45)**	**0.009**
Perception of threats from cervical cancer	1.42 (0.61 to 3.28)	0.412	0.49 (0.15 to 1.57)	0.229
Perception on medical tourism	2.25 (0.95 to 5.34)	0.064	0.91 (0.42 to 1.97)	0.819
Usage of health services in last 6 months	1.74 (0.55 to 5.45)	0.344	–	–
Societal factors^b^				
Perception of availability of prevention technologies (HPV vaccination and screening)	0.82 (0.45 to 1.50)	0.521	0.91 (0.42 to 1.97)	0.777
Perceptions of availability of equipment	0.97 (0.36 to 2.58)	0.946	0.51 (0.21 to 1.22)	0.129
Social support	0.54 (0.18 to 1.68)	0.291	0.28 (0.07 to 1.15)	0.078
Beliefs	0.19 (0.02 to 1.77)	0.145	0.70 (0.07 to 6.76)	0.758
Attitudes	1.53 (0.37 to 6.25)	0.555	0.24 (0.03 to 1.83)	0.168
Health system factors^b^				
Perception of training of Health Professionals	2.19 (0.73 to 6.60)	0.162	0.88 (0.08 to 9.32)	0.912
Perceptions of adequacy of specialists	**10.77 (3.10 to 37.32)**	**< 0.001**	**7.32 (1.53 to 35.06)**	**0.013**
Quality of care	–	**–**	0.19 (0.03 to 1.45)	0.110
Satisfaction	–	**–**	**27.15 (1.61 to 458.85)**	**0.022**
Accessibility of health facilities^c^				
Distance from nearest health facility				
Less than 10 km	1.46 (0.13 to 16.64)		1.24 (0.29 to 5.26)	0.775
21 to 40 km	Ref		Ref	–
Mode of transport to nearest health facility				
Walking	**0.08 (0.03 to 0.24)**	0.759	0.21 (0.03 to 1.80)	0.155
Public transport	0.75 (0.13 to 4.51)	–	0.21 (0.02 to 1.93)	0.169
Private car	Ref		Ref	–
Time to travel to nearest health facility		**< 0.001**		
30 or less minutes	0.87 (0.31 to 2.43)	0.015	1.50 (0.29 to 7.70)	0.624
31 to 60 min	–	–	0.83 (0.12 to 5.63)	0.848
90 or more minutes	Ref	0.794	Ref	–
		-		

^a^For healthy women, proxy indicator of access to treatment was used based on their perceptions of whether they would access treatment services for cervical cancer if diagnosed. ^b^Model 1 controlled for disease stage for patients and religion and ethnicity for healthy women, ^c^ Model 2 controlled for financial barriers for patients and religion and ethnicity for healthy women. Bold shows factors that are significant (*p* < 0.05)

Table 3 shows that the mean age of health workers was 37 years (SD = 10), with majority of health workers being below 50 years of age (92%). Average number of years of experience in health profession was 12 years (SD = 10). Fifty-four percent (54%) of the respondents were from Parirenyatwa hospital, 33% from Harare Hospital, 12% from Island Hospice and 1% from Cancer Centre. In multivariate analysis, after controlling for all factors (Table 3), perceptions of access to cervical cancer treatment from health worker’s perspective were associated with their basic training institution (*p* = 0.046), health service quality perceptions (*p* = 0.035) and electricity supply status in their respective health facilities (*p* = 0.036). However, challenges faced in seeking treatment by patients were slightly insignificant (*p* = 0.066).

**Table 3:** Description of health system attributes and factors associated with perceived access to cervical cancer treatment and care from health worker surveys

Participant type	Health worker [*N*=80]		
Variables	n (%)	Bivariate analysis *p*-value	Logistic regression analysis *p*-value
Mean age of health workers	37 (SD=10)		
23 - 30	15 (19)	0.947	-
31 – 40	42 (54)	0.162	
41 – 49	15(19)	0.947	
54+	6 (8)	**0.015**	
Mean number of years of experience of health workers			
1 – 5	12 (SD=10)		
6 – 10	22 (28)	0.469	
11 – 20	27 (34)	**0.046**	-
23+	20 (26) 9 (12)	0.432 **0.002**	
Health facilities in the survey			
Parirenyatwa Hospital	42 (54)		
Harare Hospital	26 (33)	-	-
Island Hospice	9 (12)		
Cancer Centre	1 (1)		
Continuous Professional Development support			
Yes	62 (80)		
No	8 (10)		
Not applicable	8 (10)	0.306	-
Institutions of basic training			
University of Zimbabwe	17 (22)		**0.046**
National University of Science and Technology	3 (4)	0.214	0.959
Ministry of Health and Child Welfare	58 (74)		Ref
Specialization			
Yes	41 (53)	0.645	
No	37 (47)		-
Adequacy of health professionals			
Yes	9 (11)		
No	67 (86)	0.360	
Do not know	2 (3)		-
Motivation			
Yes	54 (69)		
No	18 (23)	0.161	
Not applicable	6 (8)		-
Remuneration satisfaction			
Yes	5 (6)		
No	67 (86)	0.497	
Not applicable	6 (8)		-
Relationship with patients			
Excellent	31 (40)		
Good	44 (56)	0.592	
Poor	2 (3)		-
Refused to comment	1 (1)		
Knowledge of national cancer policy			
Yes	30 (38)	0.132	0.422
No	48 (62)		Ref
Knowledge of cervical cancer policy			
Yes	30 (38)	**0.049**	0.456
No	48 (62)		Ref
Adequacy of policies for treatment of cervical cancer			
Yes	44 (56)		0.693
No	34 (44)	**0.026**	Ref
Support treatment seeking abroad			
Yes	58 (74)	0.432	
No	20 (26)		-
Disease presentation			
Early	3 (4)	0.496	
Late	75 (96)		-
Service quality perceptions			
Excellent	22 (28)		
Good	46 (59)	0.613	**0.035**
Poor	8 (10)		
Do not know	2 (3)		
Screening services at health facility			
Yes	9 (12)	**0.002**	
No	69 (88)		-
Strength of surveillance system for cervical cancer			
Yes	18 (23)		
No	57 (73)	**0.008**	
Do not know	3 (3)		-
Adequacy of basic equipment^a^			
Yes	45 (58)		
No	27 (34)	0.984	
Do not know	6 (8)		-
Modern equipment adequacy^a^			
Yes	40 (51)	**<0.001**	0.591
No	27 (35)		
Do not know	11 (14)		
Functional equipment^a^			
Yes	36 (46)	0.633	
No	34 (44)		
Do not know	8 (10)		-
Electricity challenges^a^			
Yes	7 (9)	**0.001**	**0.036**
No	52 (67)		
Do not know	19 (24)		
Water challenges^a^			
Yes	38 (49)	**0.009**	0.674
No	24 (31)		
Do not know	16 (20)		
Cancer drug stock-outs^a^			
Yes	7 (9)	**0.001**	0.143
No	28 (36)		
Do not know	43 (55)		
Analgesic adequacy^a^			
Yes	39 (50)	0.773	
No	13 (17)		
Do not know	26 (33)		-
Analgesic stock-outs^a^			
Yes	17 (22)	0.203	0.639
No	34 (43)		
Do not know	27 (35)		
Challenges faced in seeking treatment			
Finances	29 (37)		**0.066**
Transport	49 (67)	**0.078**	
Knowledge of clinical guidelines for cervical cancer			
Yes	59 (75)		
No	13 (17)		
Do not know	6 (8)	0.757	

^a^Outputs from model 2, bold show significance or close (*p* < 0.05). Outcome variable was perception of access to treatment and care by health workers

### B. Qualitative results

#### Access to cervical cancer treatment and care

Qualitative interviews and FGDs suggested that most cervical cancer patients were not able to access treatment and care services from health facilities due to multidimensional barriers. This is shown by what some participants had to say:

“….*because we must remove the barriers and one of the barriers is cost and the other one is accessibility*”. (Senior Gynaecologist from Harare Hospital, key informant)

*“At Karanda hospital they did a biopsy after 2 weeks and her results were out and then she was referred to Parirenyatwa hospital and when we came in March (2018) the doctors were on strike*….”(Caregiver from Goromonzi,23 years)

#### Cost of diagnosis and treatment

Quantitative results from the community and patient surveys revealed that affordability was not associated with perceptions of access to treatment or uptake of treatment. However, the majority of participants in the in-depth, FGDs and key informant interviews cited the high costs of cervical cancer treatment as the biggest barrier to accessing services. The direct costs that were reported were incurred during diagnosis, staging and treatment phases, with diagnosis and staging costs causing most of the impediments to getting treatment. One junior oncologist (key informant) reported that:

*“In terms of costs I would estimate that staging and radiation therapy is about US$2000 and chemo-radiation including staging is around US$2500”*

#### Cost of biopsies

While the collection of the biopsy samples may be done in some public hospitals, the processing of the samples and investigations are done in specialized laboratories by pathologists. Qualitative findings suggest significant barriers in accessing treatment due to lack of access to biopsy histological investigations. Most respondents reported challenges in accessing the histological investigations due to limited number of doctors who can perform the procedure, long turnaround times for results from public health laboratories and the high costs involved which presents a barrier to treatment uptake. One VIAC nurse/Midwife reported that:

*“Histology is done in private laboratories and depending on the specimen that was taken they pay from US$36 upto US$56 and one may not be able to pay for their specimen”*.

#### Cost of staging

Based on the FIGO guidelines [19], cervical cancer must be staged to inform the treatment modalities. Staging involves laboratory investigations, X-rays, ultrasound scans and at times CT scans. These procedures are mandatory before a patient can be commenced on treatment. Most respondents alluded to the challenges of accessing these services due to high costs, limited availability and the time it takes to get all the investigations together for a decision to be made on a treatment plan. This was revealed by one patient in the statement below:

“….*I paid for scan and x-rays which were US$20 and US$65 respectively. When l came here l had another scan done which was US$65 and from there you realize the costs are going up”* (Patient from Kwekwe, 45 years).

#### Cost of laboratory investigations

Laboratory investigations are conducted for diagnosis, staging and during the treatment and care continuum. Some respondents reported challenges in accessing laboratory tests due to limited capacities in treating health facilities to conduct some investigations efficiently. In addition high costs are associated with laboratory tests not to mention the frequency with which they are requested before, during and after treatment. A 45 year old patient from Kwekwe reported that:

*“Blood tests were US$45 because they were done twice a week. I had 25 days for radiotherapy treatment and on the last day l was told l needed more blood and for four pints l was told it would cost me US$220 and l did not have much money left so l managed to buy only two pints*”

#### Cost of transport

Qualitative findings suggest that lack of transport or high transport costs were frequently reported barriers to accessing treatment for cervical cancer in Harare. Most of the patients treated at Parirenyatwa Hospital come from far places, outside Harare and this translated into huge transport costs. One 41 year old caregiver from Mabvuku had this to say:

*“Lack of money is also a problem because some people stay far away from hospitals so some may not even have money for transport”.*

#### Cost of palliative care related services

Our findings reveal that even palliative care services are very expensive and this burden is laid on the patients and theirfamilies. Some of the palliative care services include blood transfusion, pain medication, palliative radiotherapy and chemotherapy and visits made by palliative care staff from Island Hospice. Another 45 year old patient from Mutoko reported that:

*“When l got back (from South Africa) l went to Mutare and after four days l fell ill again and got admitted at Mutare General Hospital and I was given two pints of blood and advised to buy Ranferon tablets and some drugs to stop the bleeding and pain”*

#### Opportunity costs

Qualitative findings reveal a number of opportunity costs namely loss of ability to bear children, loss of income, loss of employment and loss of property or savings and these were mentioned by some respondents. A 49 year old Chiredzi patient reiterated that:

*“Sometimes one doesn’t go to work and has no means of getting any money to go to the hospital that is why some get to the point of dying because they don’t have money”*

#### Acceptability factors and ability to seek

Qualitative results showed a number of acceptability barriers which negatively influenced the ability to seek treatment services by patients. The most commonly cited factors were health workers’ negative attitudes, misconceptions about cervical cancer and its treatment, lack of knowledge about radiotherapy, alternative interventions (spiritual and traditional), cultural beliefs, family influences and partner attitudes or perceptions. One respondent mentioned that:

*“In our community people are very hesitant of radiotherapy as they have their own way of thinking because they don’t know what really happens. Some say the moment you undergo radiotherapy they put heavy metals on your body and your chest so your veins will not function well and they believe it is better for one to die at home than go for radiotherapy”* (Patient from Chiredzi, 49 years)

#### Approachability factors and ability to perceive

Cervical cancer treatment and palliative care services were reported by most interviewees as scarcely publicized even in health facilities. Most of the focus has been on screening and treatment of pre-cancers but there is little awareness on cervical cancer treatment. Despite some awareness campaigns on cervical cancer lack of knowledge still exists in communities and among health care workers which reduces the ability to perceive treatment services by people. Cervical cancer and its treatment are shrouded with many misconceptions that were reported by many respondents. One 39 year old cervical cancer survivor from Mabvuku mentioned that:

*“….like I mentioned earlier on, it is because of lack of awareness because cancer doesn’t bring symptoms at an early stage so one will be thinking that health wise they are ok.”*

#### Availability and accommodation and ability to reach

Many patient respondents reported travelling great distances to access treatment in Harare at Parirenyatwa Hospital. Some of the interviewees reported being put on long waiting lists for treatment while some cited health worker strikes and radiotherapy machine breakdowns as barriers to their accessing treatment. These affect the availability of treatment and patients’ ability to reach treatment facilities. A WHO official (key informant) reported that:

*“I was discussing with the director about chemotherapy in Harare that he went there and saw hundreds of women waiting and they were waiting for one machine, which breaks down here and there”*

#### Appropriateness factors and ability to engage

Qualitative results showed that there is no follow-up system for cervical cancer patients in the treatment and care continuum. Even during diagnosis stages, patients may present at many different health facilities as “new patients” to get second opinions or just an act of denial of their cervical cancer diagnosis. The health system relies on patients presenting themselves at the treating facilities for diagnosis, staging and treatment or to get follow-up reviews after their treatments. Some respondents reported missing their treatment/procedures due to challenges mostly lack of finances, beliefs, misconceptions or family influences. All these factors negatively affect the patient’s ability to engage with healthcare services. One key informant from Harare Hospital explained that:

*“The problem that we have is that if we suspect a patient has cervical cancer we may see her last the day that we would have suspected her of cervical cancer, that follow up routine is not coming out to say the person we have referred to our outpatient department (OPD) for us to know that the patient has had a biopsy taken and their results. We tell them to come back with their health cards but they don’t come back.”* (VIAC nurse from Harare Hospital, key informant)

## Discussion

There is considerable evidence that access and usage of cervical cancer treatment and palliation care services is a challenge and therefore uptake is low. Data from surveys revealed that 69% of cervical cancer patients interviewed at health facilities were on treatment while 80% of healthy women in communities perceived that they would access treatment if diagnosed of cervical cancer. These figures are consistent with what was reported in 2007 where 77% of diagnosed cervical cancer patients were treated with radiation [5]. While these figures seem high and perhaps encouraging, what remains unknown is the proportion of women with cervical cancer who are not presenting at health facilities for treatment. Furthermore, our qualitative findings suggest a myriad of barriers to treatment. These barriers are multidimensional and most patients do not get to the point of accessing histological diagnosis/confirmation or staging due to supply and demand side barriers. Our findings are supported by some recent studies which reported that patients with advanced disease were less likely to pursue further investigations for staging and some out rightly refused treatment [19–21].

Our findings revealed that no socio-demographic factors were associated with receipt of treatment. These findings are supported by other studies in similar context [20–23]. However, our qualitative research revealed financial barriers as the major hindrances to accessing diagnosis, staging and treatment services. Having medical aid was also reported by some respondents as a facilitating factor to accessing healthcare services, though there was no statistical significance in our study. These findings suggest that health system factors (few treating centres, lack of infrastructure, lack of commodities such as drugs, limited number of radiotherapy machines, frequent breakdowns of radiotherapy machines, high costs of services, few specialists, lack of standardized guidelines, lack of health information system, lack of patient follow-up system and bureaucratic referral system) and social factors (lack of knowledge, fear, stigma, misconceptions, family influences, attitudes, beliefs, influence of traditional and spiritual healers) may play a major role in treatment uptake compared to individual factors. Other researchers found that the cost of medication prescribed for cervical cancer was one of the barriers to adherence [24]. In the USA, young women with no health insurance and low socioeconomic status were likely to delay treatment for breast cancer [20]. In another study the researchers found no association between affordability (individual factor) and uptake of cervical cancer screening [25].

Knowledge of causes of cervical cancer was negatively associated with treatment uptake however; it was positively associated with perception of access to treatment among healthy women. Qualitative findings showed that more factors than knowledge seem to influence treatment utilization and these may include beliefs, attitudes, misconceptions about cervical cancer and its treatment, economic factors, health system factors, family, traditionalists’ and spiritualists’ influences. In addition, our qualitative findings suggest that those women who knew more about cervical cancer had presented late and had more knowledge because of their experiences with advanced disease. In addition, there are a number of misconceptions around radiation therapy which may present as barriers to patients especially with advance disease. One of the misconceptions is that radiation therapy quickens death hence some patients refuse this treatment modality until they have advanced disease when they become desperate. This is supported by findings in a Moroccan study that showed that patients with advanced disease were more likely to refuse further investigations and treatment itself [26]. These factors do not affect healthy women as they did not have the disease yet and the some of the factors that affect sufferers were not present. Some researchers reported that patients with lack of knowledge were at risk of delay for treatment [22]. Knowledge of prevention did not influence either treatment uptake or perception of access to it suggesting that there is little awareness of prevention of cervical cancer. Our qualitative results suggested that there are limited awareness campaigns about cervical cancer prevention and some health workers are also ignorant about these issues hence cannot educate communities effectively. Lack of knowledge about cervical cancer as a preventable disease was also reported in our qualitative interviews and focus group discussions as evidenced by misconceptions that cancer is a “*death sentence*” and that it is caused by evil spirits hence could not be prevented or treated. These findings are consistent with those of other studies that have shown that knowledge, availability or lack of it influenced access to healthcare services [20–24, 26].

This study revealed that locus of control with regards to cervical cancer was positively associated with uptake of treatment among patients but was not associated with perceptions of access to treatment among healthy women. Some researchers have defined locus of control as to the extent to which an individual perceives events and actions in his or her life as a consequence of their own behaviour, ability or characteristics (internal control) [27]. Another study reported associations between internal locus of control and uptake of cervical cancer screening [28]. Lack of association between locus of control and perceptions of access to cervical cancer could be explained by lack of knowledge and the mere of absence of disease in the respondents. Qualitative findings suggest that locus of control is crucial to early seeking treatment behaviours and uptake of treatment given the barriers associated with cervical cancer treatment. Our survey results showed no influence of societal factors on receipt of treatment and perceptions of access to cervical cancer treatment. However; qualitative findings suggested that societal factors play a significant role in the form of beliefs, attitudes, misconceptions, social support from families and partners, family influences and influence of alternative interventions’ providers (traditional healers and prophets). Most patients who reported receiving treatment had the support of their families, partners and caregivers. Our findings are supported by other similar studies [25–32].

Perceptions of adequacy of specialists were positively associated with uptake of cervical cancer treatment and perceptions of access to it among healthy women. The provision of health services rely on having a health workforce. Some studies have reported that sufficiency and distribution of health workforce is crucial in improving access to healthcare. Lack of trained health workers compromises the quality of services that can be provided [27]. Health worker survey findings suggested shortage and sub-optimally distributed health workers for the treatment and care of cervical cancer. In a South African survey, researcher found that affordability and other patient level factors (acceptability, accommodation, and accessibility) were less important predictors of access to cervical cancer screening than availability of physicians in the population [25]. Our qualitative findings revealed shortages of oncologists, oncology nurses, gynecologists, radiographers, pathologists and pharmacists for the provision of quality services. Lack of knowledge among healthcare workers about case definition and management of suspected cervical cancer was another barrier to accessing early treatment. A Malawian study reported that health workers not formally trained in cervical cancer prevention were unlikely to be responsive, fair and efficient to achieve the best outcomes [31]. This finding is consistent with our qualitative results. The Zimbabwean government has been unable to hire and train more health workers due to limited fiscal space in the last few years and this has been affecting treatment and care of cervical cancer patients [1, 2].

Our findings indicated that service satisfaction was correlated with receipt of treatment services among cervical cancer patients. This means that most patients who were treated were satisfied with the services they were provided. These findings could be due to few treatment centers in Zimbabwe to compare with and also the fact that most patients present late with severe symptoms of bleeding and pain and the treatment they are given which is usually palliative is perceived as satisfactory. Our results revealed that most respondents had reported that to them treatment meant alleviation of their pain and symptoms even if the primary condition was not treatable. The general perception was that cancer is untreatable and it is a “*death sentence*” such that any attempt to treat symptoms mostly pain and bleeding (palliative care) were well received despite the barriers they countered. All patients who received treatment had gone through many processes to access treatment and by the time they were engaged into treatment their hope was low. Some studies have shown that satisfaction with cervical cancer services was associated with knowledge of visual inspection with acetic acid (VIA) screening test, with women who knew about this procedure *apriori* being less satisfied when they were tested. Distance to the health facilities was also found to be associated with level of satisfaction with women who travelled more than five kilometers reporting higher satisfaction levels [32]. These findings are consistent with our present work with regards to satisfaction as most patients travelled great distances to get treatment at Parirenyatwa Hospital, of the major treating center in Zimbabwe.

Walking as a means of reaching nearest health facilities was negatively associated with perceptions of access to treatment for cervical cancer among healthy women. In a US study transport to health facilities was found to influence access to cervical cancer screening in urban settings [33]. Results from our qualitative study revealed that the second most frequently reported barrier to treatment was transport and its associated costs as most patients had to travel to treating facilities. Perceptions of access to cervical cancer treatment from health worker’s perspective were associated with their basic training institution, health service quality perceptions and electricity supply status in their respective health facilities. Challenges faced in seeking treatment by patients were slightly significant to perceptions of access to treatment. These findings are supported by our qualitative results which suggested that health system factors were more important to accessing treatment by cervical cancer patients.

This study deserves the justice of mentioning its limitations; that it was conducted in Harare and some findings may not be generalizable as cancer services are fairly available in Harare compared to other areas across the country. Secondly, this study comprised of cross sectional surveys whose findings may not be used to infer causal relationships. Thirdly, survey data was collected amidst a series of strikes by health workers, therefore selection bias may have been incurred as a particular group of cervical cancer patients may have visited the study sites during the study period. Lastly, the use of qualitative inquiry is inherently associated with bias and therefore the findings from the inquiry may not be generalizable beyond the study setting. This study had its fair share of strengths, to our knowledge this is the first primary research study to investigate the determinants of access and usage of cervical cancer services in Zimbabwe. The majority of studies cited have used either qualitative or quantitative methods and for policy recommendations mixed methods provide better outcomes [34, 35]. A plethora of studies in low-to-middle income countries have focused on primary and secondary prevention of cervical cancer but there is a general paucity of information on tertiary interventions in these contexts.

## Conclusions

There are numerous prevailing multi-dimensional barriers to accessing cervical cancer treatment and palliative care in Zimbabwe. Our findings revealed that heath system and societal factors are more important than individual level factors. Strategies to subsidize or remove user fees for the diagnosis, staging and treatment of cancer may go a long way to improve access to treatment in a country where the majority of people are living in poverty. Health education and promotion interventions cannot be underestimated to address the societal factors impeding treatment while reinforcing facilitating factors such as social support. Overall, multi-sectoral approaches are recommended to address all the multifaceted barriers in order to improve cervical cancer treatment and palliative care access for better outcomes in resource-limited contexts.

## Data Availability

The datasets used and/or analyzed during the current study are available from the corresponding author on reasonable request.

## Abbreviations

FGD: Focus Group Discussion
FIGO: International Federation of Gynaecology and Obstetrics
SD: Standard deviation
VIAC: Visual inspection with acetic acid cervicography
WHO: World Health Organization
ZDHS: Zimbabwe demographic and health survey
